# Cyclin-Dependent Kinases 8 and 19 Regulate Host Cell Metabolism during Dengue Virus Serotype 2 Infection

**DOI:** 10.3390/v12060654

**Published:** 2020-06-17

**Authors:** Molly Butler, Nunya Chotiwan, Connie D. Brewster, James E. DiLisio, David F. Ackart, Brendan K. Podell, Randall J. Basaraba, Rushika Perera, Sandra L. Quackenbush, Joel Rovnak

**Affiliations:** 1 Department of Microbiology, Immunology and Pathology, College of Veterinary Medicine and Biomedical Sciences, Colorado State University, Fort Collins, CO 80523, USA; molly.butler@colostate.edu (M.B.); nunya.chotiwan@umu.se (N.C.); connie.brewster@colostate.edu (C.D.B.); James.Dilisio@rams.colostate.edu (J.E.D.); David.Ackart@colostate.edu (D.F.A.); Brendan.Podell@ColoState.EDU (B.K.P.); Randall.Basaraba@ColoState.EDU (R.J.B.); rushika.perera@colostate.edu (R.P.); sandra.quackenbush@colostate.edu (S.L.Q.); 2 Arthropod-borne Infectious Disease Laboratories, College of Veterinary Medicine and Biomedical Sciences, Colorado State University, Fort Collins, CO 80523, USA

**Keywords:** dengue virus, CDK8, CDK19, hexokinase, LC3, Senexin, viral replication, transcription

## Abstract

Dengue virus infection is associated with the upregulation of metabolic pathways within infected cells. This effect is common to infection by a broad array of viruses. These metabolic changes, including increased glucose metabolism, oxidative phosphorylation and autophagy, support the demands of viral genome replication and infectious particle formation. The mechanisms by which these changes occur are known to be, in part, directed by viral nonstructural proteins that contact and control cellular structures and metabolic enzymes. We investigated the roles of host proteins with overarching control of metabolic processes, the transcriptional regulators, cyclin-dependent kinase 8 (CDK8) and its paralog, CDK19, as mediators of virally induced metabolic changes. Here, we show that expression of CDK8, but not CDK19, is increased during dengue virus infection in Huh7 human hepatocellular carcinoma cells, although both are required for efficient viral replication. Chemical inhibition of CDK8 and CDK19 with Senexin A during infection blocks virus-induced expression of select metabolic and autophagic genes, hexokinase 2 (HK2) and microtubule-associated protein 1 light chain 3 (LC3), and reduces viral genome replication and infectious particle production. The results further define the dependence of virus replication on increased metabolic capacity in target cells and identify CDK8 and CDK19 as master regulators of key metabolic genes. The common inhibition of CDK8 and CDK19 offers a host-directed therapeutic intervention that is unlikely to be overcome by viral evolution.

## 1. Introduction

According to the World Health Organization, dengue virus (DENV) is endemic in over 100 countries throughout tropical and subtropical regions worldwide. They estimate that 500,000 people with severe dengue require hospitalization each year, with an estimated 2.5% fatality rate [1]. The Pan American Health Organization reported 2,733,635 cases in 2019 in Central and South America, the highest number of cases ever recorded in the Americas; 22,127 of these were classified as severe dengue and 1206 people died [2].

Viruses depend on the host cell to provide energy and metabolic precursors to support genome replication and infectious particle production. Viral replication places increased demands for production of ATP and metabolites on the host cell [3,4,5]. To meet these demands, viruses manipulate the intracellular environment of the host cell by remodeling cellular structures and by reprogramming cellular metabolism to increase the expression of key metabolic enzymes [6,7,8,9,10,11,12,13,14].

Infections with dengue viruses are the most common mosquito-borne diseases worldwide and pose a significant risk to approximately half of the world’s population [15,16]. There are four distinct serotypes, dengue 1, 2, 3, and 4 (DENV1–4), each causing a similar, acute disease marked by fever and severe joint pain. Sequential infections with different DENV serotypes are associated with dengue hemorrhagic fever (DHF) and dengue shock syndrome (DSS), which are life threatening in 15% of patients [17,18]. Of the four serotypes, DENV2 has been shown to rely on increased glucose uptake and enhanced glucose metabolism to provide necessary metabolic intermediates for viral replication [7,8,19]. DENV2 infection also induces autophagy and lipophagy for efficient replication [11,20,21,22,23,24]. Lipophagy releases fatty acids from lipid droplets which are shuttled into mitochondria for β-oxidation and ATP production [11,22,24]. Recent work has identified interactions between viral non-structural proteins and host metabolic enzymes to modify these processes [10,19,25]. Beyond protein–protein interactions, it has also been shown that expression of mRNAs encoding specific metabolic enzymes is elevated during DENV2 infection, and this increases the total metabolic capacity of infected cells [8,9]. We sought to identify host factors that regulate these transcriptional changes in metabolic enzyme expression during DENV2 infection.

Cyclin-dependent kinase 8 (CDK8) is a component of the general transcription factor, Mediator, a large protein complex required for transactivation of all RNA polymerase II (RNA Pol II) transcription. CDK8 regulates transcriptional elongation by phosphorylation of the C-terminal domain of the largest subunit of RNA Pol II, a variety of transcription factors, and serine 10 on histone H3 (H3S10) [26,27,28,29,30]. CDK8 regulates gene expression preferentially during induced states such as hypoxia and serum starvation [27,28,31,32] and has recently been implicated in the transcription of glycolytic and autophagic genes [33,34].

We have previously shown that CDK8 is directly and very specifically targeted by the oncogenic retrovirus, walleye dermal sarcoma virus [31,32,35,36], and reasoned that CDK8 is likely targeted, directly or indirectly, by many viruses with metabolic and autophagic demands, as exemplified by DENV2 infection. We found that expression of CDK8 is upregulated during DENV2 infection of Huh7 human hepatocellular carcinoma cells, and that this precedes increased expression of two key genes, hexokinase 2 (HK2) and microtubule-associated protein 1 light chain 3 (LC3). Knockdown of expression of CDK8, the CDK8 paralog, CDK19, or their activator, Cyclin C, reduced viral genome replication. CDK8 and CDK19 share identical active sites, which allows their specific inhibition with small-molecule competitive inhibitors. We used the active-site inhibitor, Senexin A and its second-generation iteration, Senexin B [37,38]. These compounds reduced DENV2 induction of HK2 and LC3 expression, viral RNA replication, and the production of infectious particles. Inhibition of CDK8/CDK19 kinase activity also reduced mitochondrial function in infected and uninfected cells. The results identify DENV2 dependence on CDK8 function to regulate host gene expression, a host mechanism that can be targeted for therapeutic interdiction.

## 2. Materials and Methods

### 2.1. Cell Culture and DENV2 Infection

Huh7 cells, a hepatocellular carcinoma cell line derived from a liver tumor in a 57-year-old Japanese male in 1982 (https://huh7.com), were provided by Dr. Rushika Perera and cultured in Dulbecco’s modified Eagle medium (DMEM), supplemented with 10% fetal bovine serum (FBS), 1% non-essential amino acids, 1% L-glutamine, 1% penicillin-streptomycin, and 25 mM HEPES. At 24 h prior to infection, 1 × 10^6^ Huh7 cells were plated in 25 cm^2^ flasks. Immediately prior to infection, cells were incubated at 4 °C for 15 min, washed with cold Dulbecco’s phosphate-buffered saline (D-PBS) and incubated with DENV2 (strain 16681, passage 4) [39] at 4 °C for 1 h with rocking. Virus media were removed and replaced with supplemented DMEM with 2% FBS.

### 2.2. CDK8/19 Small-Molecule Inhibition and Cell Viability Assay

Mock- or DENV2 infected cells were incubated in DMEM with Senexin A (MedChemExpress, Monmouth Junction, NJ, USA) or Senexin B (ProbeChem, Shanghai, China) at indicated concentrations or in media with the volumetric equivalent (final 0.01%) of DMSO solvent for the indicated period of time. Cell viability with Senexin treatment or shRNA transduction was determined by modified MTS assay according to the manufacturer’s instructions (CellTiter 96 Aqueous One Solution Cell Proliferation Assay, Promega, Madison, WI, USA).

### 2.3. qRT-PCR Analysis

At the indicated times post infection or treatment, cells were collected in TRIzol (Invitrogen, Thermofisher, Waltham, MA, USA), and total RNA was isolated according to the manufacturer’s instructions and treated with Turbo DNase I (Ambion-Life Technologies, Thermofisher, Waltham, MA, USA). cDNA was synthesized with an iScript cDNA synthesis kit (Bio-Rad, Hercules, CA, USA) according to the manufacturer’s instructions, and then, subjected to qPCR analysis with iQ SYBR green Supermix in a CFX96 real-time PCR system (Bio-Rad, Hercules, CA, USA). Primer sequences are listed in Table 1. Relative expression was normalized to the housekeeping gene, Succinate Dehydrogenase Complex Flavoprotein Subunit A, *SDHA* [40]. For genomic equivalent analysis, Cq values were standardized to ten-fold dilutions of in vitro transcribed DENV2 genomic RNA and subject to qRT-PCR.

### 2.4. Plaque-Forming Unit and Extracellular Genome Equivalent Analysis

Media were collected from virus-infected cells at indicated times, centrifuged at 500× *g* for 15 min to remove cellular debris, and aliquoted into TRIzol LS (Invitrogen, Thermofisher, Waltham, MA, USA). RNA was extracted according to the manufacturer’s instructions, and cDNA was synthesized with an iScript cDNA synthesis kit (Bio-Rad, Hercules, CA, USA) and subjected to qPCR analysis with iQ SYBR green Supermix in a CFX96 real-time PCR system (Bio-Rad, Hercules, CA, USA). Cq values were compared to ten-fold dilutions of in vitro transcribed DENV2 genomic, as described above.

Plaque assays were performed on BHK cells. Briefly, 10-fold dilutions of clarified supernatant were adsorbed on confluent BHK cells for 1 h. The cells were then overlaid with 3 mL of 1% agarose in MEM supplemented with 5% FBS. After incubation for 8 days, 4% neutral red solution in PBS was added to the agar overlay, and plaques were counted at 18–24 h after staining.

### 2.5. Lentivirus-Mediated shRNA Gene Knockdown

Lentivirus delivery of short hairpin RNAs (shRNA) (Sigma-Aldrich, St. Louis, MO, USA; listed in Table 2) was used to knock down expression of CDK8, CDK19, and Cyclin C. A non-target shRNA was used as a control. 293FT cells (Thermofisher, Waltham, MA, USA) were transfected with shRNA and lentivirus packaging constructs, and virus particles were collected after 48 h. Huh7 cells were transduced with shRNA lentiviruses at an MOI of 1 and incubated for 48 h prior to selection with 1 µg/mL puromycin for four days. Selected cells were harvested for protein assay or replated at 1 × 10^6^ cells per 25 cm^2^ flask for DENV2 infection (MOI = 1) for 24 h.

### 2.6. Subcellular Extracts and Protein Analysis

Nuclear extracts were prepared as previously described [35]. Briefly, cells were lysed in 0.5% NP-40 in PBS with protease and phosphatase inhibitors (2 µg/mL leupeptin and aprotinin, 1 µg/mL pepstatin, 0.2 mM phenylmethylsulfonyl fluoride [PMSF], 0.2 mM sodium orthovanadate, 2 mM sodium pyrophosphate, and 1 mM glycerophosphate). Nuclei were pelleted at 1500× *g*, washed with cold PBS, and then, with buffer A (10 mM HEPES pH 8.0, 10 mM KCl, 1.5 mM MgCl_2_, 0.5 mM dithiothreitol (DTT)), prior to extraction overnight with 2.5 nuclear pellet volume buffer C (10 mM HEPES pH8.0, 420 mM KCl, 20% glycerol, 0.1 mM EDTA, 0.5 mM DTT, and protease and phosphatase inhibitors). Extracted nuclei were pelleted for 15 min at 21,000 g and re-extracted with 1 pellet volume buffer C. Extracts were pooled for analysis. Remaining chromatin-bound proteins were prepared from extracted nuclear pellets after an additional wash with buffer C and equilibration in DNAse digestion buffer (100 mM Tris HCl, pH 7.5, 25 mM MgCl_2_, and 5 mM CaCl_2_) prior to addition of digestion buffer with 1 Unit DNAse I /µL and rotation at 4 °C for 72–96 h. Digested chromatin preparations were clarified at 21,000 g for 15 min.

Mitochondrial extracts were prepared as previously described [46]. Briefly, cells were swelled in cold hypotonic buffer (10 mM NaCl, 1.5 mM MgCl2, 10 mM Tris-HCl pH7.5) and broken in a Dounce homogenizer. Lysates were brought to 210 mM mannitol, 70 mM sucrose, 5 mM Tris-HCl (pH 7.5) and 1 mM EDTA with 2.5X homogenization buffer. Nuclei were removed by centrifugation at 1300× *g* for 5 min, and then, mitochondria were pelleted at 15,000 g for 15 min, washed once in homogenization buffer and suspended in immunoprecipitation (IP) buffer (1% Triton X-100, 0.5% NP-40, 150 mM NaCl, 10 mM Tris-HCl (pH 7.5), 1 mM EDTA (pH 8.0), 1 mM EGTA, and protease and phosphatase inhibitors).

Protein concentration of each extract was determined with a Pierce BCA protein assay kit (Thermofisher, Waltham, MA, USA) according to the manufacturer’s instructions. Equal quantities of total protein were separated by polyacrylamide gel electrophoresis for western blot. Blocked blot segments, separated by molecular weight range, were probed simultaneously with indicated primary antibodies (Table 3 and Table 4; GeneTex, Irvine, CA, USA; Novus Biologicals, Littleton, CO, USA; Cell Signaling Technology, Danvers, MA, USA; Santa Cruz Biotechnology, Santa Cruz, CA, USA; Molecular Probes, Thermofisher, Waltham, MA, USA; Proteintech, Rosemont, IL, USA) overnight. Antibodies were detected with appropriate horseradish peroxidase-conjugated secondary antibodies and developed with the TMB membrane peroxidase substrate system (3,3′,5,5′-Tetramethylbenzidine, Seracare Life Sciences, Milford, MA, USA). Images were scanned with a Visioneer One Touch 9420 scanner (Visioneer, Pleasanton, CA, USA) at a gamma value of 1.0, and all contrast adjustments were uniformly applied using Adobe Photoshop (Adobe, Inc., San Jose, CA, USA). High contrast images were measured using NIH ImageJ gel analysis software (Version 1.53b, National Institutes of Health, Bethesda, MD, USA) to determine band densities.

### 2.7. Mitochondrial and Glycolytic Stress Tests

A Seahorse XFe analyzer (Agilent Technologies) was used to measure the oxygen consumption rate (OCR) and extracellular acidification rate (ECAR) based on the mitochondrial stress test (Agilent 103015-100) and glycolysis stress test (Agilent 103020-100), respectively. Huh7 cells were plated in XF96 cell culture microplates at 2 × 10^4^ cells per well 16 h prior to infection with DENV2 at an MOI of 10. At 24 or 48 hpi, the culture media were replaced with 180 µL of XF base medium (Agilent Technologies) supplemented with 1 mM pyruvate, 2 mM L-glutamine, and with 10 mM glucose for the mitochondrial stress test or without glucose for the glycolysis stress test. The cells were rested for one hour in a non-CO_2_ incubator at 37 °C, then placed in the Seahorse for analysis. The mitochondrial stress test used sequential injections of oligomycin (1 µM), p-trifluoromethoxyphenylhydrazone (FCCP, 1.5 µM), and rotenone and antimycin A (0.5 µM each). The glycolysis stress test used sequential injections of glucose (10 mM), oligomycin (1 µM), and 2-deoxyglucose (50 mM). Measurements were collected at 5 minute intervals; three times before and after injections and six times after the last injection. After analysis, cell numbers were measured by addition of 22 µL 10 nM Calcein-AM to all wells and absorption read at 520 nm after excitation at 488 nm in a SpectraMax M2^e^ plate reader (Molecular Devices). Calcein readings were imported into Wave (Agilent) software to normalize OCR and ECAR readings.

Mitochondrial activity was determined as follows: (1) Basal respiration was calculated using the last rate measurement before injection of oligomycin minus the non-mitochondrial respiration rate (defined as the minimum rate measurement after rotenone/antimycin A injection), (2) ATP production was calculated as the last rate measurement before oligomycin injection minus the minimum rate measurement after oligomycin injection, (3) Maximum respiration was calculated using the maximum rate measurement after FCCP injection minus non-mitochondrial rate, (4) Spare capacity was the maximal respiration minus basal respiration, and (5) Proton leak was the minimum rate measurement after oligomycin injection minus non-mitochondrial respiration. Glycolytic function was determined as follows: (1) Glycolysis was the maximum rate measurement before oligomycin injection minus the last rate of measurement before glucose injection, (2) Glycolytic capacity was the maximum rate measurement after oligomycin injection minus the last rate of measurement before glucose injection, (3) Glycolytic reserve was the glycolytic capacity minus glycolysis and (4) Non-glycolytic acidification was the last rate measurement prior to glucose injection.

### 2.8. Statistics

Statistical analysis was performed on Prism software (Version 8.1.2 (227)).

## 3. Results

### 3.1. Cyclin-Dependent Kinase 8 Is Upregulated during DENV2 Infection

**Figure 1:**
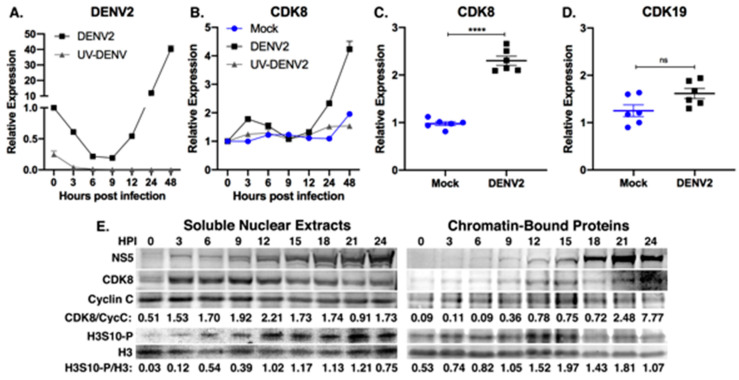
CDK8 is upregulated during DENV infection. (**A**,**B**) Huh7 cells were either mock-infected, infected with DENV at MOI 10, or treated with equivalent UV-inactivated DENV2 (UV-DENV) over a time course of 48 h. Total cellular RNA was collected at indicated time points after infection and analyzed by qRT-PCR for DENV RNA (**A**), and CDK8 mRNA expression (**B**), relative to the time of infection and normalized to the housekeeping gene, *SDHA*. Results are representative of two independent experiments. (**C**,**D**) CDK8 (**C**) and CDK19 (**D**) mRNA expression in mock or DENV-infected Huh7 cells after 36 h of infection at MOI 10 (n = 6 biological replicates; **** *p* < 0.0001 unpaired, two-tailed *t* test). Error bars represent mean +/− SEM). (**E**) Western blot analyses of 10 µg of total protein from nuclear extracts (Soluble Nuclear Extracts) and from the remaining, salt-extracted chromatin fraction (Chromatin-Bound Proteins) from cells collected every three hours for 24 h after infection. Antibody specificities are indicated as are the relative band densities of CDK8 versus Cyclin C and histone H3 phosphorylated at serine in position 10 (H3S10-P) versus total histone H3 (H3). Infection was confirmed by presence of nuclear DENV2 NS5. The results are representative of three separate time-course infections.

To investigate the role of CDK8 during DENV2 replication, we quantified CDK8 mRNA over time after synchronized infection with a high multiplicity of infection (MOI) to reach complete or near-complete infection of all cells in the culture, and mitigate effects from uninfected bystander cells. Huh7 cells were mock-infected, infected at an MOI of 10, or treated with a matched preparation of UV-inactivated DENV2 particles (UV-DENV2). After binding at 4 °C, cells were washed and incubated in media at 37 °C. Total RNA was harvested at 0, 3, 6, 9, 12, 24 and 48 h post infection (hpi) and virus replication was monitored by qRT-PCR (Figure 1A). We observed a modest increase in CDK8 mRNA levels in infected cells at 3 hpi followed by a steady rise after 12 hpi, coincident with maximal virus replication (Figure 1A,B). We saw the sharpest increase in CDK8 mRNA expression between 24 and 48 hpi in DENV2-infected cells relative to mock-infected cells and cells treated with UV-inactivated virus (Figure 1B), and subsequently observed a 2.3-fold increase in CDK8 mRNA levels at 36 hpi in six biological replicates (Figure 1C; mean = 2.30 +/− 0.09, *p* < 0.0001). A 2.3-fold increase in CDK8, while modest, may have a profound reprogramming effect on the host cell due to the cascading nature of CDK8-mediated transcriptional regulation. Viral RNA from cells treated with UV-DENV2 was taken up by cells (Figure 1A), but did not result in viral RNA replication or increased expression of CDK8 (Figure 1B), demonstrating that the induction of CDK8 expression is dependent upon uptake of infectious DENV2. In contrast to the increase in CDK8 expression, we found no significant change in CDK19 mRNA expression (Figure 1D), suggesting a specific demand for increased CDK8 during DENV2 infection. CDK8 and its paralog CDK19 are highly conserved in their kinase and cyclin-binding domains, but have unique C-terminal domains, suggesting similar but divergent functions [47,48].

CDK8 protein levels were also increased in DENV2-infected cells compared to the start of infection (Figure 1E). Increased CDK8 was visible in nuclear extracts at 3 hpi, coincident with the first appearance of DENV2 nonstructural protein 5 (NS5) in the nuclei of infected cells (Figure 1E). Apparent CDK8 levels in the soluble nuclear fraction remained elevated, relative to Cyclin C, throughout the course of infection and were coincident with a progressive increase in the phosphorylated form of a known CDK8 substrate, H3S10-P [29,49], relative to total histone H3 (Figure 1E). In addition to increased CDK8 in the salt-soluble nuclear fraction, CDK8 in the remaining chromatin fraction began to increase after 6 hpi (Figure 1E, see methods for details). Increased chromatin-bound CDK8 was also coincident with increasing NS5 levels in the chromatin fraction. The partition of DENV2 NS5 protein between soluble nuclear and chromatin fractions has been observed previously [50,51,52,53]. There was also an apparent increase in H3S10-P, relative to H3 levels, in the chromatin fraction (Figure 1E). H3S10-P is a mark of active, open chromatin [29,49].

These data demonstrate that CDK8 mRNA and protein levels increase after DENV2 infection and suggest active CDK8 phosphorylation of histone H3 at serine 10 in association with increased transcriptional activity. CDK8 chromatin occupancy is also increased during DENV2 infection, an outcome associated with enhanced CDK8-dependent gene expression [27,28,31,32].

### 3.2. Knockdowns of CDK8 and CDK19 Reduce DENV2 Replication

**Figure 2:**
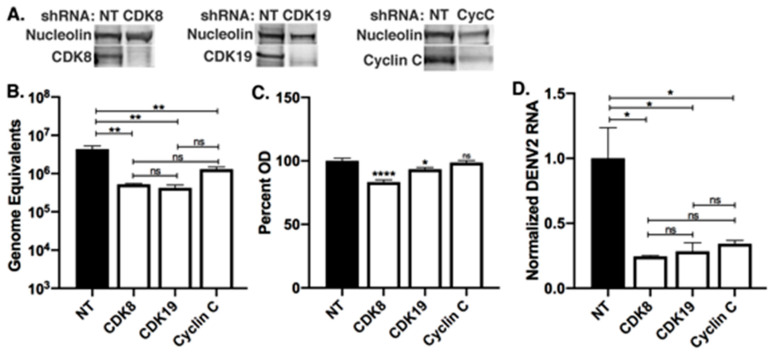
Knockdowns of CDK8 and CDK19 reduce DENV2 replication. (**A**) Western blot analysis of 10 µg of total protein from nuclear extracts of Huh7 cells transduced at an MOI of 1 with lentivirus-mediated non-target control or CDK8-targeted shRNA, CDK19-targeted shRNA, or cyclin C-targeted shRNA. (**B**) Lentivirus-transduced Huh7 cells were infected with DENV2 at MOI 1 for 24 h, and total cellular RNA analyzed by qRT-PCR for DENV RNA quantification relative to in vitro transcribed DENV genome equivalent (GE) standard curve (*n* = 3 biological replicates per group; ** *p* < 0.01; one-way ANOVA with Tukey’s multiple comparisons test. Error bars represent mean +/ SEM). (**C**) Relative optical density read in lentivirus-transduced Huh7 cells after four days of selection, then treated with CellTiter 96 Aqueous One Solution. (*n* = 4 biological replicates. * *p* < 0.05, **** *p* < 0.0001; one-way ANOVA with Dunnett’s test. Error bars represent mean +/ SEM). (**D**) Lentivirus-transduced Huh7 cells were infected with DENV2 at MOI 1 for 24 h, and total cellular RNA analyzed by qRT-PCR for DENV RNA quantification normalized to the housekeeping gene, *SDHA*, and relative to expression in non-target controls (*n* = 3 biological replicates per group; * *p* < 0.05, one-way ANOVA with Tukey’s multiple comparisons test. Error bars represent mean +/ SEM).

To investigate the roles of CDK8 and CDK19 during DENV2 infection, we utilized lentivirus-mediated shRNA knockdowns of CDK8, CDK19 and their activating cyclin, Cyclin C, in Huh7 cells (Figure 2A). Huh7 cells were transduced with non-target-shRNA (NT-shRNA) or CDK8-shRNA, selected in puromycin for four days, and then, mock-infected or infected with DENV2. For the purpose of evaluating changes in efficiency of viral replication and transmission within the cell culture, we elected to use an MOI of 1 and quantitated DENV2 genome equivalents (GE) and DENV2 RNA relative to housekeeping gene transcripts in infected cells. Knockdown of CDK8 reduced DENV2 replication 8.5-fold when compared to NT-shRNA controls (NT-shRNA mean = 4.4 ± 0.9 × 10^6^ GE vs. CDK8-shRNA mean = 5.2 ± 0.3 x10^5^ GE, *p* = 0.002), and CDK19 knockdown reduced replication 10.5-fold (CDK19-shRNA mean= 4.2 ± 0.8 × 10^5^ GE, *p* = 0.002) (Figure 2B).

Knockdown of either CDK8 or CDK19 has previously been shown to reduce the proliferation rate of cultured cells [31,54,55], and we observed reduced proliferation of CDK8 and CDK19 knockdowns, compared to NT-shRNA control cells (Figure 2C), so we normalized DENV2 RNA expression to a housekeeping gene to correct for reduced cell numbers. There was a significant decrease in DENV2 RNA in CDK8 and CDK19 knockdown cells relative to cellular RNA (Figure 2D). Knockdown of Cyclin C (CycC-shRNA) expression (Figure 2A), which activates both CDKs and is a proxy for CDK8/19 knockdown [26], did not affect cell numbers but did reduce DENV2 replication 3.3-fold (NT-shRNA mean = 4.4 ± 0.9 × 10^6^ GE vs. CycC-shRNA mean = 1.3 ± 0.2 × 10^6^ GE, *p* = 0.009) (Figure 2B–D).

### 3.3. CDK8/19 Chemical Inhibition Reduces DENV2 Replication

**Figure 3:**
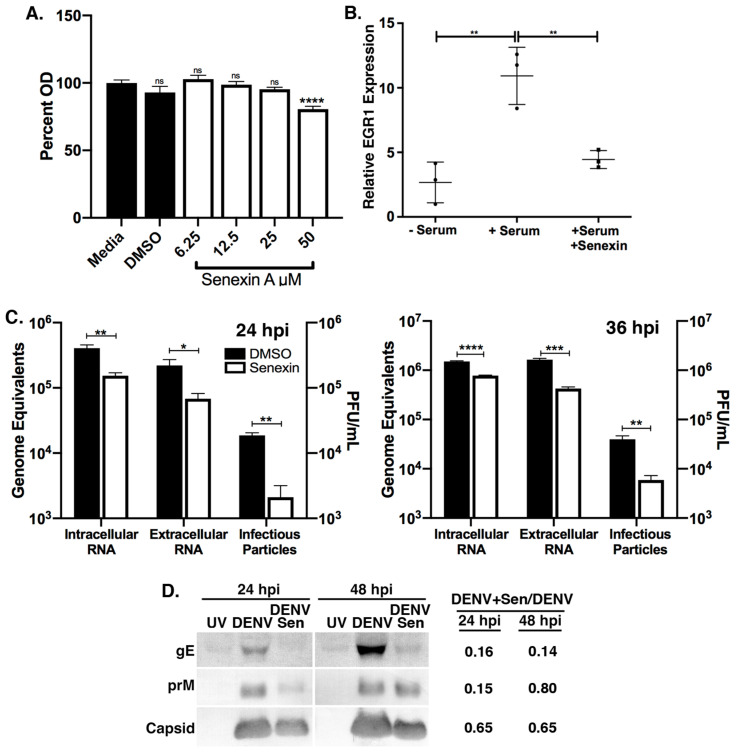
CDK8/19 Chemical inhibition reduces DENV2 replication. (**A**) Relative optical density of Huh7 cells after 72 h treatment with DMSO or Senexin A at indicated doses and addition of CellTiter 96 Aqueous One Solution. (*n* = 9 biological replicates. **** *p* < 0.0001 one-way ANOVA with Dunnett’s test. Error bars represent mean +/ SEM). (**B**) *EGR*1 mRNA levels in serum-starved and serum-stimulated Huh7 cells in the presence of DMSO or 12 μM Senexin A (n = 3 biological replicates; * *p* < 0.05, **** *p* < 0.0001 one-way ANOVA with Dunnett’s test; error bars represent mean +/− SEM). (**C**) DENV2 RNAs in total cellular RNA preparations (Intracellular RNA, GE) and in culture supernatants (Extracellular RNA, GE) were determined by qRT-PCR, and supernatant virus measured by plaque assay (Infectious Particles). Huh7 cells were infected with DENV2 (MOI = 1) for 24 or 36 hpi with DMSO or 12 or 25 μM Senexin A, respectively (*n* = 3 biological replicates; * *p* < 0.05, ** *p* < 0.01, *** *p* < 0.001, **** *p* < 0.0001; unpaired two-tailed *t* test; Error bars represent mean +/ SEM). (**D**) Western blot assays of supernatant virus pellets from cells infected with 10 MOI UV-treated DENV2 (UV), or with DENV2 without (DENV) or with 25 µM Senexin A (DENV/Sen) at 24 and 48 hpi. Antibody specificities are indicated. Relative band densities of DENV2 gE, prM, and capsid proteins in DENV2-infected, Senexin A-treated versus DENV2-infected virus preparations are indicated to the right of the indicated panels. Results are representative of three biological replicates for each time point.

To better study CDK8 and CDK19 function during DENV2 infection, we utilized a small-molecule CDK8/19 active site inhibitor, Senexin A [38]. The use of Senexin A does not distinguish CDK8 and CDK19 activities, as their active sites are identical, but Senexin A treatment can be administered exclusively during infection without prior treatment or selection and did not negatively affect Huh7 cell proliferation at the concentrations used in this study (12–25 μM) (Figure 3A). Senexin inhibition of CDK8/19, in contrast to knockdowns, exclusively inhibits kinase activity rather than eliminating both kinase- and structure-related functions of CDK8/19 [38,48]. We verified Senexin A inhibition of CDK8 function by inhibiting expression of a known CDK8-dependent serum response gene, *EGRI* [27,31] (Figure 3B). Cells which were serum-starved and subsequently serum-repleted exhibited the characteristic induction of *EGR1*, while cells treated with Senexin A did not significantly induce *EGR1* expression in response to serum (Figure 3B).

Huh7 cells were treated with dimethyl sulfoxide (DMSO) or Senexin A solubilized in DMSO at the start of synchronized infections, and virus replication was assessed at 24 hpi (after one round of virus replication) (Figure 3C) and at 36 hpi (coincident with significantly increased CDK8 expression, Figure 1C and Figure 3D). At 24 hpi, 12μM Senexin A reduced intracellular virus RNAs 2.7-fold compared to DMSO controls (DMSO mean = 4.1 ± 0.5 × 10^5^ GE vs. Senexin A mean = 1.5 ± 0.2 × 10^5^ GE, *p* = 0.008) (Figure 3C). Senexin A treatment also reduced extracellular virus RNAs 3.2-fold (DMSO mean = 2.2 ± 0.5 × 10^5^ GE vs. Senexin A mean = 6.8 ± 1.4 × 10^4^ GE, *p* = 0.04) and infectious virus particles by 8.9-fold (DMSO = 1.9 ± 0.2 × 10^4^ PFU/mL vs. Senexin A mean = 2.1 ± 1.0 × 10^3^ PFU/mL, *p* = 0.0015) (Figure 3C). At 36 h hpi, 25 μM Senexin A reduced DENV2 intracellular virus RNAs 1.9-fold (DMSO, 1.5 × 10^6^ GE vs. Senexin A, 7.8 × 10^5^ GE), extracellular RNAs by 3.8-fold (DMSO, 1.6 × 10^6^ GE vs. Senexin A, 4.2 × 10^5^ GE) and PFU/mL by 6.8-fold (DMSO, 4.0 × 10^4^ vs. Senexin A, 5.9 × 10^3^) (Figure 3C).

We analyzed the viral proteins in Senexin A-treated cells by western blot (Figure 3D). Cell-free virus particles, pelleted from equal volumes of infected-cell supernatants, showed reduction in envelope protein, E, prM and capsid levels (Figure 3D). Although levels of capsid protein were outside of the linear range of the western analysis, the relative band densities of E at 24 and 48 hpi and of prM at 24 hpi in supernatants from Senexin A-treated cells have approximately 15% of the protein in the untreated cells. The results from Senexin A inhibition of CDK8/19 during DENV2 infection indicate a significant deficit in the synthesis of viral RNA and in the production and packaging of infectious viral particles.

### 3.4. Metabolic Gene Expression Is Dependent on CDK8/19 Kinase Activity

**Figure 4:**
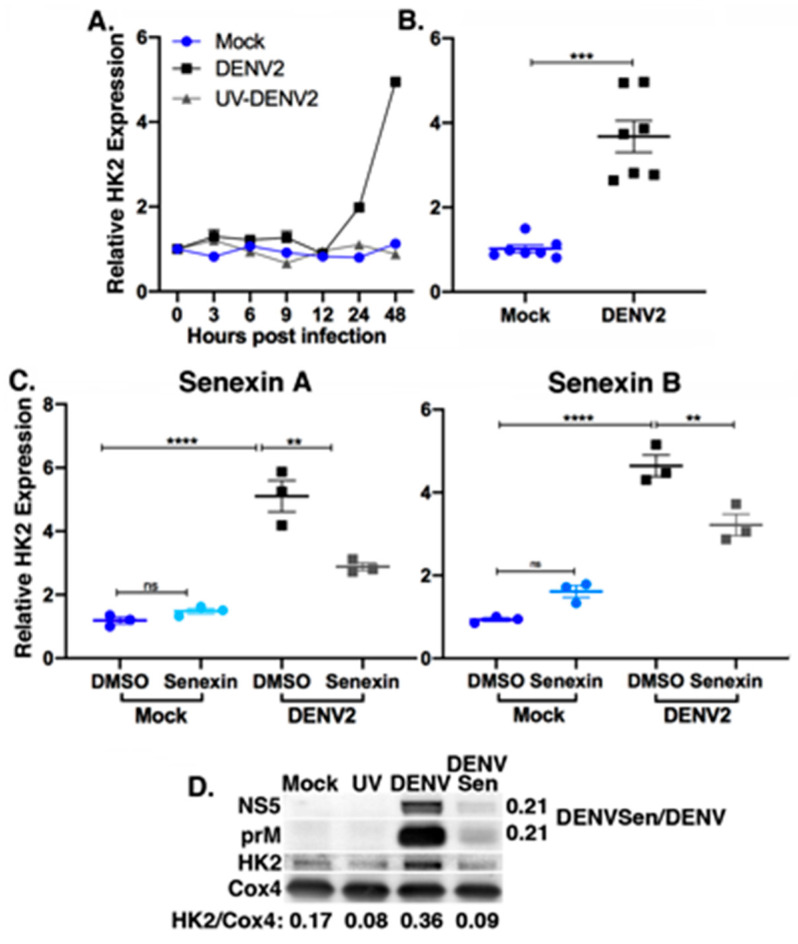
Senexin A reduces DENV2 induction of Hexokinase 2 expression. (**A**) Huh7 cells were either mock-infected or infected with DENV at MOI 10, and HK2 mRNA expression analyzed by qRT-PCR at indicated time points. Results are representative of two independent experiments. Expression is relative to time of infection and normalized to *SDHA*. (**B**) HK2 expression in mock vs. infected cells at 48 hpi (*n* = 7 biological replicates; *** *p* < 0.001 unpaired, two-tailed *t* test with Welch’s correction). Error bars represent mean +/− SEM. (**C**) HK2 mRNA expression in mock-infected and infected Huh7 cells treated with DMSO or 25 μM Senexin A or 12 μM Senexin B added at start of infection (MOI 10; 36 hpi). Expression relative to mock-infected, DMSO-treated cells and normalized to *SDHA* (*n* = 3 biological replicates; ** *p* < 0.01, **** *p* < 0.0001; one-way ANOVA with Tukey’s multiple comparisons test). Error bars represent mean +/− SEM. (**D**) Western blot analysis of cellular and viral protein abundance in 2 µg mitochondrial-enriched fractions from mock-infected cells (Mock), or cells with 10 MOI UV-treated DENV2 (UV) or DENV2 without (DENV) or with or 25 µM Senexin A (DENV/Sen). Antibody specificities are indicated as are the relative band densities of HK2 versus Cox4. Relative band densities of DENV2 NS5 and prM proteins in Senexin A-treated versus DMSO-treated, DENV2-infected cell preparations are indicated to the right of the indicated panels. Results are representative of six biological replicates.

CDK8 kinase activity has been shown to control the transcription of select glycolytic genes [33]. We investigated the role of CDK8 and CDK19 as regulators of the metabolic pathways that are induced during DENV2 infection. The early steps in glycolysis are critical for production of metabolites that support DENV2 replication and are marked by an increase in glucose uptake and increased expression of the first rate-limiting enzyme in glycolysis, HK2 [7,8]. We measured HK2 mRNA expression during the time course of synchronized DENV2 infections and confirmed that it is upregulated compared to mock-infected cells and cells infected with equivalent UV-inactivated DENV2 (Figure 4A). Increased HK2 mRNA levels were detected after 12 hpi, coincident with maximal virus RNA replication and with increased CDK8 mRNA expression (Figure 1 A–C). HK2 mRNA levels were increased 3.68-fold (±0.38, *n* = 7) in DENV-infected Huh7 cells at 48 hpi (Figure 4B).

We assessed the impact of Senexin A on HK2 expression during synchronized DENV2 infections and observed inhibition of the virus-induced HK2 expression but not of basal HK2 expression. Senexin A did not entirely block HK2 induction by infection but did reduce it (2.88 ± 0.21-fold increase over mock vs. a 5.10 ± 0.49-fold increase in DMSO-treated infected cells; Figure 4C). Mock-infected cells showed no difference in HK2 expression with or without Senexin A treatment (Figure 4C). While conducting these experiments, we obtained a second generation CDK8/19 inhibitor, Senexin B [37], and confirmed a similar 2.1-fold reduction in DENV2 genome equivalents with Senexin B treatment as we observed with Senexin A (DMSO GE mean = 6.7 ± 1.0 × 10^7^, Senexin B GE mean = 3.2 ± 0.7 × 10^7^). As with Senexin A treatment, Senexin B inhibited the induction of HK2 during DENV2 infection, while basal expression of HK2 was unaffected (Figure 4C, right panel; DMSO mean fold change over mock: 4.64 ± 0.45, Senexin B: 3.22 ± 0.45).

In addition to HK2, we evaluated mRNA levels of four other glycolytic genes which have been shown to be responsive to CDK8 function: phosphofructokinase (PFK), glyceraldehyde 3-phosphate dehydrogenase (GAPDH), enolase isoform 1 (ENO1), and pyruvate kinase isoform M2 (PKM2) [33]. Expression of these four genes was not upregulated during DENV2 infection of Huh7 cells (data not shown). This is in line with previous work demonstrating an anapleurotic role for HK2 in the production of metabolic intermediates rather than an ATP-producing role [7,8].

HK2 localizes to the outer mitochondrial membrane, where it functions in the regulation of glucose metabolism, protection from apoptosis, and initiation of autophagy [8,56,57,58]. We prepared mitochondria-enriched fractions [46], which are also known to be enriched in viral replication complexes and DENV2 proteins [59]. We observed increased HK2, relative to levels of a mitochondrial marker protein, cytochrome *c* oxidase subunit 4 (Cox4), in preparations from DENV2-infected cells compared to mock and UV-DENV2-treated cells, and Senexin A treatment reduced HK2 abundance to levels in uninfected cells (Figure 4D). Senexin treatment also reduced the apparent levels of NS5 and prM in these preparations to 21% of levels in DENV2-infected cell preparations (Figure 4D). Cox4 protein levels were apparently unaffected by Senexin treatment.

Overall, these data indicate that Senexin inhibition of CDK8/19 kinase activity specifically reduces virus-induced but not basal levels of HK2 mRNA and protein expression, and that the expression of the host protein, Cox4, is unaffected by both DENV2 infection and by Senexin A treatment.

### 3.5. Senexin A Reduces Induction of Lipophagic Gene Expression

In addition to changes in glucose utilization, DENV2 induces lipophagy, the autophagic depletion of lipid droplets for release of free fatty acids to support a metabolic shift to β-oxidation [11]. LC3 functions in the formation of the autophagosomal membrane on lipid droplets [60]. LC3 is initially modified to LC3-I by the autophagy factor Atg4, and subsequently modified to the membrane-associated, lipidated form, LC3-II, by autophagy factors Atg3 and Atg7 [61]. Conversion of LC3-I to LC3-II is considered a measure of autophagic activity [60,61], and increased conversion of LC3-I to LC3-II has been observed during DENV2 infection [23,24].

**Figure 5:**
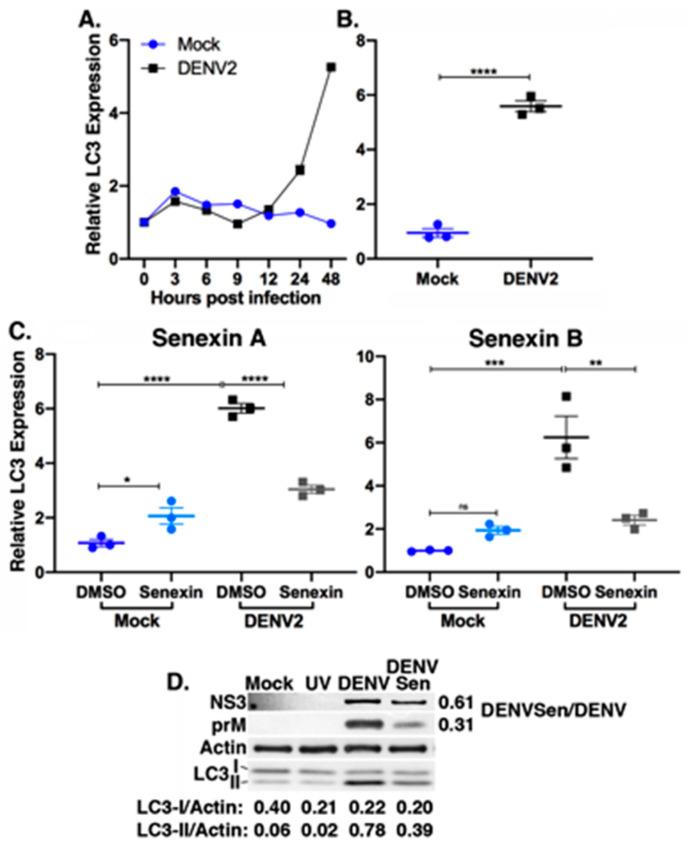
Senexin A reduces DENV2 induction of lipophagic gene expression. (**A**) Huh7 cells were either mock-infected or infected with DENV at MOI 10, and LC3 mRNA expression analyzed by qRT-PCR at indicated time points. Expression relative to time of infection, normalized to *SDHA* and representative of two independent time course experiments. (**B**) LC3 expression in mock vs. infected cells at 48 hpi (*n* = 3 biological replicates; *** *p* < 0.001 unpaired, two-tailed *t* test with Welch’s correction). Error bars represent mean +/− SEM. (**C**) LC3 mRNA expression in Huh7 cells mock-infected or infected with DENV2 at MOI 10 for 36 h with DMSO or 25 μM Senexin A or 12 μM Senexin B added at time of infection (relative to mock-infected, DMSO-treated cells and normalized to *SDHA*; *n* = 3 biological replicates. * *p* < 0.05, ** *p* < 0.01, *** *p* < 0.001, **** *p* < 0.0001; one-way ANOVA with Tukey’s multiple comparisons test). Error bars represent mean +/− SEM. (**D**) Western blot analysis of cellular and viral protein abundance in 2 µg cytoplasmic extracts from mock-infected cells (Mock), or cells with 10 MOI UV-treated DENV2 (UV) or DENV2 without (DENV) or with or 25 µM Senexin A (DENV/Sen). Antibody specificities are indicated as are the relative band densities of LC3-I and LC3-II versus Cox4. Relative band densities of DENV2 NS3 and prM proteins in Senexin A-treated versus DMSO-treated, DENV2-infected cell preparations are indicated to the right of the indicated panels. Results are representative of six biological replicates.

We found that the level of LC3 mRNA increased over the course of DENV2 infection coincident with the increases in CDK8 and HK2 (Figure 1, Figure 4 and Figure 5A). LC3 mRNA levels increased 5.6 ± 0.2-fold (*p* < 0.0001) in DENV2-infected versus mock-infected cells at 48 hpi (Figure 5B). As with HK2 (Figure 4), the viral induction of LC3 mRNA expression was significantly reduced in cells treated with Senexin A (DMSO mean fold change over mock: 6.02 ± 0.31, Senexin A: 3.04 ± 0.26) and with Senexin B (DMSO mean fold change over mock: 6.25 ± 1.70, Senexin B: 2.41 ± 0.38) (Figure 5C). In addition, as observed with HK2 expression, basal LC3 expression was not inhibited by CDK8/19 inhibition. Senexin A, but not Senexin B, actually induced a modest increase in LC3 expression in mock-infected cells (Figure 5C).

Western blot analyses of cytoplasmic extracts demonstrated a consistent increase in lipidated LC3-II in DENV2-infected cells compared to mock-infected or UV-DENV2-treated cells, relative to β actin levels (Figure 5D). Senexin A treatment reduced levels of lipidated LC3-II by half (Figure 5D). As observed previously, Senexin A treatment also reduced virus protein levels in cellular extracts. In this case, NS3 was 61% and prM 31% of that observed in untreated, DENV2-infected cells. Levels of host actin protein remained unaffected by treatment with Senexin A.

Overall, these data indicate that Senexin inhibition of CDK8 and CDK19 kinase activity specifically reduces infection-induced but not basal levels of LC3 gene expression, and that the expression of host proteins Cox4 (Figure 4D) and actin (Figure 5D) is unaffected by both DENV2 infection and by Senexin treatment.

### 3.6. Senexin A Inhibits Mitochondrial Respiration

**Figure 6:**
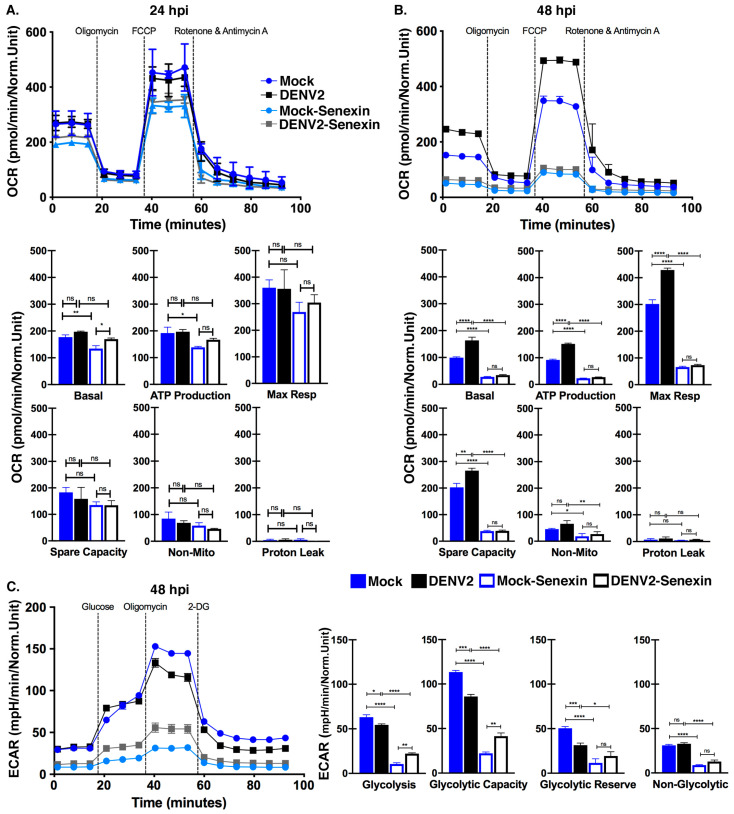
Senexin A inhibits mitochondrial respiration. Huh7 cells were either mock-infected or infected with DENV2 at an MOI of 10 with or without DMSO or 12.5 µM Senexin A for 24 or 48 h. (**A**,**B**) Normalized oxygen consumption rate (OCR) was measured over time during mitochondrial stress tests. Specific values determined by the mitochondrial stress test (Basal rate, ATP production, Maximum Respiration, Spare Capacity, Proton Leak, and Non-Mitochondrial Respiration) are presented. (**C**) Normalized extracellular acidification rate (ECAR) was measured over time during a glucose stress test at 48 hpi. Specific values determined in the glucose stress test (Glycolysis levels, Glycolytic Capacity, Glycolytic Reserve, and Non-Glycolytic Capacity) are presented. Results were normalized to viable cell numbers after metabolic measurements. Parameters for individual measurements are presented in the methods (* *p* < 0.05, ** *p* < 0.01, *** *p* < 0.001, **** *p* < 0.0001 one-way ANOVA with Tukey’s multiple comparisons test. *n* = 3 biological replicates). Error bars represent mean +/ SEM.

We directly tested the downstream consequences of DENV2 infection and CDK8/19 inhibition by measuring metabolic flux in mock and DENV2-infected Huh7 cells with and without Senexin A treatment. We used a Seahorse XF metabolic flux analyzer to measure the rates of mitochondrial respiration as indicated by oxygen consumption rate (OCR) following sequential addition of oligomycin (ATP synthase inhibitor), FCCP (uncoupler of oxidative phosphorylation) and rotenone and antimycin A (electron transport chain complex I and III inhibitors) (Figure 6) (see methods for details regarding calculations of parameters).

We first evaluated changes in metabolic capacity in DENV2-infected compared to uninfected Huh7 cells. At 24 hpi, there were no significant differences in rate of oxygen consumption between uninfected and DENV2-infected cells (Figure 6A; top panel: measurements over time; bottom panels: quantification, see methods for details regarding time points for quantification). However, at 48 hpi, there were substantial increases in basal respiration, ATP production, maximal respiration, and spare capacity in DENV2-infected cells compared to mock-infected cells (Figure 6B; top panel: measurements over time, bottom panels: quantification). Only proton leak and non-mitochondrial respiration were unchanged in DENV2-infected cells. These data are in line with our host gene expression data, supporting an increase in metabolic capacity of DENV2-infected Huh7 cells compared to uninfected cells.

We evaluated differences in oxygen consumption between uninfected and DENV2-infected cells with or without Senexin A treatment to determine if CDK8/19 kinase activity plays a role in the metabolic changes during DENV2-infection. Senexin A reduced basal respiration and ATP production in mock-infected cells after 24 h, but the differences in other parameters and in DENV2-infected cells were not significant (Figure 6A). In contrast, we observed significant changes in metabolic flux with Senexin A treatment at 48 hpi (Figure 6B). All the parameters of respiration except proton leak were reduced and there were no longer distinguishable differences between DENV2-infected and uninfected cells (Figure 6B). Therefore, extended Senexin A inhibition of CDK8/19, from 24 to 48 h, resulted in a profound suppression of mitochondrial respiration, coincident with reduced metabolic gene expression (Figure 4 and Figure 5).

We also evaluated changes in glycolysis during DENV2 infection, as increased glucose uptake has been previously reported [8]. If increased glucose uptake in DENV2-infected cells was utilized for ATP production, we would expect to see increased extracellular acidification due to the production of lactic acid. We measured the extracellular acidification rate (ECAR) in uninfected and DENV2-infected cells following sequential addition of glucose, oligomycin (ATP synthase inhibitor) and 2-deoxy-glucose (2-DG) (hexokinase inhibitor) (see methods for details regarding calculations of parameters). As evidenced by diminished ECAR, glycolysis, glycolytic capacity and glycolytic reserve were all significantly reduced by DENV2 infection (Figure 6C; left panel: measurements over time, right panels: quantification). This supports the conclusion that glucose metabolism during DENV2 infection does not serve an ATP-producing role, but functions in the production of metabolic intermediates [7].

We also evaluated Senexin A’s effects on glycolytic capacity. Senexin A treatment reduced glycolytic and non-glycolytic capacity in both mock- and DENV2-infected samples (Figure 6C). However, unlike mitochondrial respiration, cells infected with DENV2 were able to compensate, modestly, for the effects of Senexin A on glycolysis and glycolytic capacity (Figure 6C). Together, these data illustrate the dependence of cellular metabolism on CDK8 and/or CDK19 kinase activity and show that this dependence may be manipulated by DENV2 to support the increased metabolic demands required for efficient viral replication.

## 4. Discussion

Here we show that expression of CDK8, a highly conserved transcriptional regulator, is upregulated during DENV2 infection, suggesting a role for CDK8 in the cellular response to infection. Increased levels of CDK8 are coincident with increased expression of two key metabolic genes, *HK2* and *LC3*, which support DENV2 replication. Manipulation of the host cell environment is critical to support the demands of viral replication, and alterations in metabolism have been the focus of a number of recent papers identifying host factors necessary for DENV replication [6,7,9,62,63]. Autophagy is also a common outcome of flavivirus infection, and lipid droplet metabolism is linked to replication as a source of neutral lipids to meet energy demands, as a source of intermediates for de novo fatty acid synthesis, and as a platform for viral assembly in the case of hepatitis C [11,14,20,21,22,23,24,64,65,66,67,68,69,70,71,72,73]. We show that CDK8/19 kinase activity is required for the altered metabolism observed during DENV2 replication.

We first noted changes in CDK8 mRNA levels during synchronized DENV2 infections but found no significant change in expression of the CDK8 paralog, CDK19 (Figure 1). Neither mock-infected or cells with bound, UV-treated DENV2 particles showed increased CDK8, indicating a requirement for virus replication and/or virus protein production. The specific functions of CDK8 remain unclear, but likely include expression of the transcription factors that activate expression of HK2 and LC3 and other proteins that support virus replication.

We used lentivirus-mediated shRNA CDK8 and CDK19 knockdowns to distinguish roles for these transcription cofactors during DENV2 infection and found that both are necessary for robust virus replication (Figure 2). However, only CDK8 expression was significantly upregulated during DENV2 infection. This suggests only that CDK19 levels do not need to be increased to support DENV2 replication, as opposed to the clear association of infection with increased CDK8. Basal levels of CDK19 may be sufficient for replication, and the effect of CDK19 knockdown indicates that it does have a supportive role in DENV2 infection. Such a role may include function independent of its kinase activity [47,48,54]. Further investigation into unique kinase-dependent and kinase-independent roles for CDK8 and CDK19 during DENV2 infection is warranted and will provide insight into functional differences between these two mediator kinases.

Chemical inhibition of CDK8 and CDK19 with the small-molecule inhibitor Senexin A reduced viral genome replication and infectious particle formation (Figure 3). CDK8/19 chemical inhibition also reduced the expression of HK2 and LC3 in infected cells to near mock-infected levels (Figure 4 and Figure 5). As expected, the outcome of the Senexin A block of HK2 and LC3 induction in DENV2-infected cells was loss of the increased mitochondrial respiration associated with infection (Figure 6). What was not expected was the significant decline in mitochondrial respiration in the Senexin A-treated, mock-infected Huh7 carcinoma cells. These data indicate that mitochondrial respiration in Huh7 cells is chronically induced and subject to CDK8/19 regulation without involvement of virus. As little as two-fold over-expression or over-active CDK8 is common in many cancerous cell types, and expanded metabolic capacity plays a role in the establishment and maintenance of many tumors [37,38,55]. Indeed, the exceptional capacity of hepatic tumor cell lines to support DENV2 replication may lie in their already expanded metabolic activity, which appears to be dependent upon CDK8/19 enzymatic activity. Though not within the scope of this work, investigations into CDK8/19-dependent metabolism in primary cells may yield valuable insight.

The data show that Senexin A has an inhibitory effect on the metabolic capacity of cells, and that this effect cannot be overridden by DENV2 infection. The delay until after 24 h for the full effects of Senexin A suggests that it is acting, as expected, at the level of gene expression; CDK8/19 inhibition blocks the gradual reprogramming of gene expression that results in substantive metabolic changes during maximal virus replication. The timing of DENV2 induction of HK2 and LC3 expression until after 12 hpi, with full induction only after 24 h, and the ability of Senexin A to block HK2 and LC3 induction, supports a role for CDK8/19 specifically in the control of gene expression to regulate metabolism.

CDK8 was recently shown to regulate autophagy in *Drosophila* under starvation conditions by promoting the elongation of mRNAs encoding *Atg1* and *Atg8*, orthologs of mammalian ULK1 and LC3 [34]. Elongation of the 3′ UTR is achieved through CDK8 phosphorylation of the cleavage and polyadenylation specificity factor, CPSF6, a component of the cleavage and polyadenylation (CPA) complex. Tang et al. [34] further showed that treatment with Senexin A reduced activation of LC3, which, in accordance with our findings, suggests a conserved role for CDK8 in the regulation of LC3, independent of cellular stressors. Loss of LC3 gene expression is a likely mechanism for reduced mitochondrial respiration in the mock- and DENV2-infected Huh7 cells treated with Senexin A. Dependence of DENV2 replication on increased mitochondrial capacity is consistent with recent reports on the expansion of the mitochondrial compartment during DENV infection [59]. Cyclin C has also been implicated in the control of mitochondrial dynamics independently of CDKs [74,75,76]. Any of these proposed mechanisms are consistent with the reductions in DENV2 RNA that we observed with the knockdown of CDK8, CDK19, and Cyclin C (Figure 2B,D). Further mechanistic investigations are warranted.

In addition to changes in mitochondrial dynamics, DENV2-infected cells exhibited reduced glycolytic activity, as previously observed, due to diversion of glycolytic intermediates from lactic acid production to anapleurotic roles [7,77]. Senexin A treatment reduced the glycolytic activity, in terms of lactic acid production, of both mock- and DENV2-infected cells (Figure 6C), indicating CDK8/19 control of glycolytic gene expression. DENV2 infection did allow a modest increase in glycolysis and glycolytic capacity compared to mock-infected cells in the presence of Senexin A. This compensation may be the result of higher levels of HK2 expression in Senexin A-treated, DENV2-infected cells compared to treated, mock-infected cells (Figure 4C) due to an incomplete Senexin A block of CDK8 in infected cells.

Though we used HK2 and LC3 as measures of CDK8/19-dependent metabolic gene expression during DENV2 infection, these are likely not the only genes regulated by CDK8/19 during viral infection. More investigation into the global picture of CDK8/19-regulated gene expression during DENV2 infection is warranted to understand their complete role. We previously showed that CDK8 activity is enhanced by a retroviral cyclin [31,32,35]. Interaction between the retroviral cyclin and CDK8 enhances CDK8 function in the serum response to the advantage of the virus, which requires cell proliferation for transmission. We now show that CDK8 is increased during infection with an unrelated virus, DENV2, and that increased CDK8 activity is ultimately beneficial to viral replication. In this case, the support is dependent on metabolic rather than proliferative mechanisms. DENV2 dependence on host metabolism makes it a valuable model to study CDK8/19 control of metabolism.

CDK8 is highly conserved across all metazoan organisms, and metabolic flux and induction of autophagy are common to infections by a broad array of DNA and RNA viruses [20,24]. We propose that many viruses may depend on CDK8 and/or CDK19 function for induced expression of metabolic and autophagic genes. The cellular demands for CDK8/19 function appear to be limited to proliferative and stress responses as opposed to homeostasis. As such, therapeutics that target CDK8/19 enzyme activity offer promise for virus infection as well as cancer [37]. We observed a reduction in PFUs of almost one log_10_ copies/mL in the supernatants of Senexin A-treated cells. Waggoner et al. [78] calculated that the odds of severe dengue disease are increased by 50% for each increase in 1 log_10_ copies /mL of acute-phase serum. Senexin therapies have the capacity to significantly reduce severe outcomes of DENV infections. In addition to significant reductions in disease severity, there is a minimum mosquito infectious dose in the serum of infected individuals. Nguyen et al. [79] quantitated the 50% mosquito infectious dose at 6.29–7.52 log_10_ RNA copies/mL of plasma; a log_10_ reduction in plasma virus that reduces viral load below this range will effectively reduce the capacity for DENV transmission. Further reductions in viral loads may be achieved with combination therapies of additional host-directed and virus targets, a common strategy to avoid the development of resistance to virus-targeted drugs alone. In summary, we have identified CDK8/19 as a hub of transcriptional regulation during DENV2 infection. CDK8 and CDK19 are responsible for the upregulation of key metabolic genes that enhance glucose metabolism and autophagy to meet the energetic and metabolic demands of viral replication.

## Figures and Tables

**Table 1:** PCR Primers.

Gene	Forward	Reverse	Source
CDK8	GGGATCTCTATGTCGGCATGTAG	AAATGACGTTTGGATGCTTAAGC	[28]
CDK19	GCCACGGCTAGGGCCT	GCGAGAACTGGAGTGCTGATAA	[28]
CyclinC	ATGGCAGGGAACTTTTGGCAG	ACCGTAGCAGTGGCAATAACT	Birkenheuer, unpublished
DENV	ACAAGTCGAACAACCTGGTCCAT	GCCGCACCATTGGTCTTCTC	[41]
HK2	CAAAGTGACAGTGGGTGTGG3	GCCAGGTCCTTCACTGTCTC3	[42]
LC3	AAGGCTTTCAGAGAGACCCTG	CCGTTTACCCTGCGTTTGTG	[43]
ENO1	GTCTCTTCAGGCGTGCAAGC	GATGAGACACCATGACGCCC	[33]
PFKL	GGCATTTATGTGGGTGCCAAAGTC	CAGTTGGCCTGCTTGATGTTCTCA	[44]
PKM2	CCACTTGCAATTATTTGAGGAA	GTGAGCAGACCTGCCAGACT	[45]
GAPDH	GCCATCAATGACCCCTTCAT	CGCTCCTGGAAGATGGTG	[35]
SDHA	GACAACTGGAGGTGGCATT	CCGTCATGTAGTGGATGGCA	[40]

**Table 2:** shRNA sequences.

Gene	Designation	Sequence
CDK8	TRCN0000000489	CCGGATGTCCAGTAGCCAAGTTCCACTCGAGTGGAACTTGGCTACTGGACATTTTTT
CDK19	TRCN0000195069	CCGGAGGACTGATAGCTCTTCTTTACTCGAGTAAAGAAGAGCTATCAGTCCTTTTTT
Cyclin C	TRCN0000020189	CCGGGCATCCAAAGTAGAGGAATTTCTCGAGAAATTCCTCTACTTTGGATGCTTTTT
Nontarget	SHC002	CCGGCAACAAGATGAAGAGCACCAACTCGAGTTGGTGCTCTTCATCTTGTTGTTTTT

**Table 3:** Cell Protein Antibodies.

Antibody	Source	Catalog Number
Rabbit anti ß Actin	Cell Signaling	4967
Goat anti CDK8	Santa Cruz Biotechnology	sc 1521
Rabbit anti Cyclin C	Novus Biologicals	NB1202950
Mouse anti Cytochrome C oxidase subunit IV	Molecular Probes	A21347
Mouse anti Phospho Histone H3 (Ser10)	Cell Signaling	9706
Rabbit anti Histone H3	Cell Signaling	9715
Rabbit anti Hexokinase 2	Proteintech	AP#22029
Rabbit anti LC3B	Novus Biologicals	NBP246892SS

**Table 4:** Virus Protein Antibodies.

Antibody	Source	Catalog Number
Mouse anti Flavivirus group antigen protein E, Clone 4G2	Novus Biologicals	NBP252079
Mouse anti DENV2 NS5 protein	GeneTex	GTX629447
Rabbit anti DENV capsid protein	GeneTex	GTX103343
Rabbit anti DENV prM protein	GeneTex	GTX128093
Mouse anti DENV NS3 protein	GeneTex	GTX124252
